# Assessing the specificity of the Rosette agent DNA amplification: An optimized protocol for the detection of standard DNA among studies

**DOI:** 10.1111/jfd.13722

**Published:** 2022-09-30

**Authors:** Emira Cherif, Theo Deremarque, Fabienne Justy, Rodolphe Elie Gozlan, Marine Combe

**Affiliations:** ^1^ ISEM, Univ Montpellier, CNRS, IRD Montpellier France

**Keywords:** disease surveillance, fish fungal parasite, intracellular parasite, nested‐PCR, ONT sequencing, *Sphaerothecum destruens*

## 1 INTRODUCTION

In 2005, a risk of global disease emergence in freshwater fish was identified in Europe and associated with the presence of the fungal‐like pathogen *Sphaerothecum destruens* (Rosette Agent) (Combe & Gozlan, 2018; Gozlan et al., 2005). *Sphaerothecum destruens* is a unicellular, intracellular obligate and generalist parasite (Andreou et al., 2011; Arkush et al., 2003) that exhibits high levels of virulence (i.e. host mortality rates) in a wide variety of freshwater and marine fish species (including at least 18 cyprinids and salmonids species) in the wild and in aquaculture facilities and has been found in a wide range of climates such as the mountainous Tcheremoch River in Ukraine to the desert wadi Felrhir in Algeria (reviewed in Combe & Gozlan, 2018). For example, in 1984 *S. destruens* was responsible for high mortalities observed in Chinook salmon (*Oncorhynchus tshawytscha*) in seawater net pens in Washington state, USA (Harrell et al., 1986). Since then, it has been responsible for two mortality events of brown and rainbow trout in farms and experimental facilities in western France (Boitard et al., 2017) and the total extinction (up to 80%–90% of mortality) of native fish populations in a catchment area in southeast Turkey (Ercan et al., 2015). Importantly, the presence of this fungal‐like parasite in such a variety of freshwater ecosystems is enhanced by the highly invasive nature of its healthy (i.e. asymptomatic) carrier the topmouth gudgeon, *Pseudorasbora parva* (reviewed in Gozlan, 2011).

The intracellular and fungal‐like nature of *S. destruens* combined with low parasite loads in healthy carriers makes it extremely difficult to detect in infected hosts and therefore introduces a bias when we estimate the disease's true prevalence in a population. Furthermore, the use of slightly different primer sets to target and amplify its DNA from complex environmental matrices (e.g. water and fish tissues) results in an underestimation of its prevalence among invasive fish populations (Combe et al., 2022) and significant variations in prevalence levels between different studies (see Combe et al., 2022; Ercan et al., 2015; Gozlan et al., 2005; Spikmans et al., 2013). After 15 years of research resulting in 28 publications and despite the high risk of disease emergence worldwide associated with irreversible impacts on fish biodiversity and the economy of the aquaculture sector, the scientific community still questions the reliability, sensitivity and efficiency of 3 different PCR primer sets commonly used to detect its DNA in healthy carriers and infected native fish species. However, the first step for effective control of the emergence and spread of the rosette agent in natural ecosystems and aquaculture facilities is a highly specific and sensitive DNA detection method that allows us to estimate the true extent of infection. Here, our aim was to provide decision‐makers and stakeholders with a more reliable detection tool. We suggest that this optimized protocol becomes the one commonly used worldwide to obtain comparable results between studies.

## 2 MATERIAL AND METHODS

**FIGURE 1:**
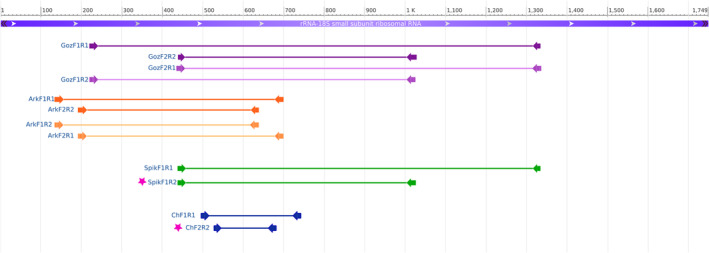
Primer combinations tested in this study. The sequence corresponds to *Sphaerothecum destruens* 18S rRNA gene. The origin of each PCR assay is indicated by the author's name: Goz = primers from Gozlan et al. (2005); Ark = primers from Mendonca and Arkush (2004); Spik = primers from Spikmans et al. (2020). Ch = newly designed primer (this study). The location on the 18S rRNA gene of the targeted region is represented by coloured lines and arrows: F, forward primer; R, reverse primer; 1: first PCR; 2: nested‐PCR. Dark colours correspond to published primer combinations. The light colours correspond to the published primers and to the new combinations tested in this study. The pink star marks the selected combination for optimized detection of *S. destruens* DNA.

First, the DNA of five fish (P12, B127, Abl76, G100, G138) previously found positive by qPCR for *S. destruens* (Combe et al., 2022) was used to test the detection efficiency of previously published primer sets. In parallel DNA from 40 fishes collected from endemic areas in France, such as Corsica, the Camargue and the Indre regions, was used to test several primer combinations and validate the best one. In total, forty fish DNA samples corresponding to nine species were analysed (Table S1). A pure *S*. *destruens* DNA (RA‐1 isolate, ATCC® 50643™) provided by the Laboratoire des Pyrénées et des Landes (LPL) was used as a positive control and DNA from parasite‐free common carp brain cells (CCB) available at the ISEM laboratory was used as a negative control. The specificity of 10 primer combinations (Figure 1; Table S2) corresponds to pre‐existing nested‐PCR assays from Gozlan et al. (2005), Mendonca and Arkush (2004) and Spikmans et al. (2020) and targeting an 18S rRNA gene sequence was tested following the author's amplification protocols. Next, the nested‐PCR products (PCR amplification products) were validated using a low‐cost amplicon‐based nanopore sequencing approach. Sequencing was performed on an Oxford Nanopore Technologies (Oxford, UK) MinION device with a runtime of 12 h. Then, for each nested‐PCR result, the bases of the raw FAST5 files with a run sequencing tag were recalled using the super‐accurate model implemented in the ONT Guppy v5.0.7 basecaller. The subsequent bioinformatic analysis included only reads between 100 and 700 bp. The size filtering step was performed by the *guppyplex* command implemented in the ARTIC pipeline (https://github.com/artic‐network/fieldbioinformatics). The filtered amplicon reads were mapped to the 18S sequence (GenBank accession: AY267345.1) of *Sphaerothecum destruens* isolate BML using minimap2 v2.17 (Li, 2018). BAM files were indexed, sorted and filtered with SAMtools v1.10 (Li et al., 2009). BAM files were filtered using the option ‘‐F' of Samtools view with the flag “4” to remove unmapped reads (Li et al., 2009). The resulting filtered BAM files were used as input to Medaka v1.0.3 (https://github.com/nanoporetech/medaka) to build a consensus for each sample. A multi‐sequence alignment step was performed between the samples and the reference sequence using MAFFT (Katoh et al., 2019). After analysing the results, an optimized PCR assay was sought. The most conserved region was identified and a new primer set was designed with Primer3Plus (Untergasser et al., 2007). The performance of the new set was evaluated by running an in silico PCR on the last release of the SILVA databases (Quast et al., 2012) using TestPrime 1.0 (Klindworth et al., 2013). Thus, a new nested‐PCR assay (Figure 1; Table S2) was developed and tested (for the detailed protocol, see https://www.protocols.io/private/D04B5C6A909711EC84BC0A58A9FEAC02).

## 3 RESULTS

All the published primer combinations tested in this study generated multiple band profiles indicating possible nonspecific co‐amplifications (Figure S1). Sequencing analyses of the PCR amplicons confirmed the hypothesis of the lack of specificity of the primers. For each combination tested, only 0.00069% to 5.45% (Table 1) of the reads mapped to the 18S ribosomal sequence of *S. destruens*. In comparison, 1.57%, 76%, and 99.72% of the positive control (pure RA‐1 isolate) reads were mapped to the targeted 18S sequence using the published primer combinations.

**TABLE 1:** Percentage of reads mapped to the 18S sequence of *Sphaerothecum destruens* for each nested‐PCR

Samples	ArkF2R1	ArkF2R2	SpikF1R2	ChF2R2
P12	–	–	5.45	89.05
G100	0.0015	NA	0.0012	0.04
G138	0.0016	0.0015	0.00046	0.32
B127	0.00076	0.01	0.0011	89.32
Abl76	0.0019	NA	0.00069	0.09
RA	1.57	76.4	99.72	*

*Note*: Ark = primers from Mendonca and Arkush (2004); Spik = primers from Spikmans et al. (2020); Ch = newly designed primer (this study); – = Not tested; * = Not sequenced; NA = No Amplification; P12 = *Pseudorasbora parva*; G100 and G138 = *Rutilus rutilus*; B127 = *Rhodeus amarus*; Abl76 = *Alburnus alburnus*; RA = positive control; Pure *S. destruens* DNA isolate RA‐1.

**FIGURE 2:**
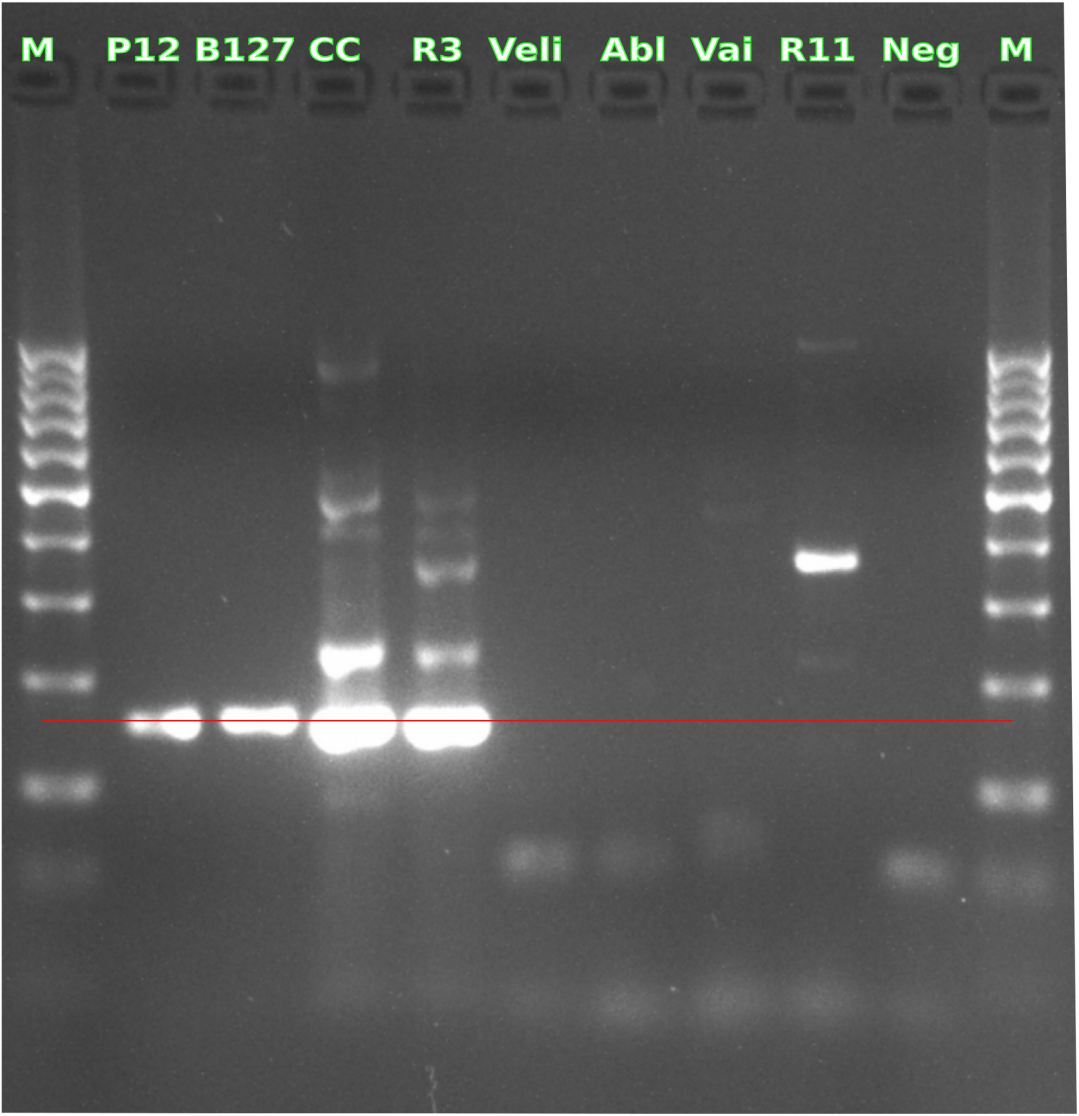
Image of an agarose gel showing detection results of *Sphaerothecum destruens* extracted from six fish species using nested‐PCR with the ChF2/ChR2 primer set. P12 = *Pseudorasbora parva*, B127 = *Rhodeus amarus*, Vai = *Phoxinus phoxinus*, Abl = *Alburnus alburnus*, CC = *Cyprinus carpio*, R3 and R11 = *Scardinius erythrophthalmus*. Veli = Cyprinus carpio brain cells Negative control. Neg = no‐template control. M = DNA Ladder. P12 is used as a positive control. The red line indicates the target band at 154 bp.

Of the nested‐PCR sequencing results, the F1R2 combination from Spikmans et al. (2020) showed higher specificity (99.72% of the positive control reads were mapped to the 18S sequence of *S. destruens*). To gain more specificity, we, therefore, developed a new PCR assay in which we combined the F1R2 primer combination of Spikmans et al. (2020) (first PCR) with the new primers (ChF2R2) designed in a highly conserved region of *S. destruens'* 18S sequence (nested‐PCR step). This approach was tested on P12 and B127 DNA samples, as well as 40 other fish DNA samples and generated multi‐ and single‐band profiles (Figure 2, Figure S2) without amplification in the pathogen‐free control. Interestingly, *S. destruens'* specificity was significantly improved >10‐fold compared with results obtained using F1R2 (Spikmans et al., 2020) for nested‐PCR. More than 89% of the reads from the P12 and B127 samples were mapped to the 18S sequence of *S. destruens* (see Table 1). Although not identified, we can hypothesize that the co‐amplification generating the multiband profiles may correspond to other regions from the unsequenced genome of the rosette agent. Nonetheless, *a primer‐fish binding* can not be entirely excluded even if the in silico PCR on SILVA databases (Quast et al., 2012) did not show matches with fish 18S rDNA. However, while greatly increasing the specificity of *S. destruens* DNA detection in infected fish for diagnostic purposes, we suggest that even this new nested‐PCR assay cannot avoid confirming the amplification of the 18S sequence of *S. destruens* by a sequencing step. In addition, tuning the annealing temperature using the “Gradient PCR” method can improve specificity. On the other hand, the isolation and sequencing of the entire *S. destruens* genome will be of paramount importance in the development of new markers for even more sensitive and specific sequencing‐free parasite detection, especially for low‐load samples.

## 4 CONCLUSION

By combining Nested‐PCR and amplicon‐based nanopore sequencing, we have developed a more specific and reliable diagnostic test for the disease. This method should be used as a standard detection protocol *S. destruens* to monitor its emergence in natural ecosystems and aquaculture facilities. More importantly, this method is an essential addition to the toolbox of epidemiological approaches to invasion.

## CONFLICT OF INTEREST

The authors declare that they have no conflict of interest.

## Supporting information


Appendix S1

## Data Availability

Data are available from the authors upon reasonable request.
