# Supplementary material for: Assessing the specificity of the Rosette agent DNA amplification: An optimized protocol for the detection of standard DNA among studies
Source: J Fish Dis. 2022 Sep 30;46(2):177–80. doi: 10.1111/jfd.13722 (PMC10092284; doi:10.1111/jfd.13722)
Supplement: Supplementary file 1 — Appendix S1 [file JFD-46-177-s001.docx]

**Table S1** List of DNA samples used in this study to perform the different nested-PCR assays

| **Sample Name** | **Scientific Name** | **Sampling Location** | **Year of Sampling** |
| --- | --- | --- | --- |
| P12 | *Pseudorasbora parva* | La Chalaronne, Ain, France | 2017 |
| B127 | *Rhodeus amarus* | La Claise, Indre, France | 2017 |
| Abl76 | *Alburnus alburnus* | Fumemorte, Camargue, France | 2019 |
| G138 | *Gobio gobio* | Périssac, Gironde, France | 2018 |
| G100 | *Rutilus rutilus* | Périssac, Gironde, France | 2018 |
| R1-R18 | *Scardinius erythrophthalmus* | Calacuccia, Corse, France | 2020 |
| T1-T6 | *Salmo trutta* | Calacuccia, Corse, France | 2020 |
| P1-P5 | *Pseudorasbora parva* | Calacuccia, Corse, France | 2020 |
| Vai | *Phoxinus phoxinus* | Calacuccia, Corse, France | 2020 |
| P1-P6 | *Pseudorasbora parva* | Fumemorte, Camargue, France | 2020 |
| CC | *Cyprinus Carpio* | Fumemorte, Camargue, France | 2020 |
| Per | *Perca fluviatilis* | Fumemorte, Camargue, France | 2020 |
| Abl | *Alburnus alburnus* | Fumemorte, Camargue, France | 2020 |
| Veli | *Cyprinus Carpio,* brain cells Negative control | I-SEM laboratory | 2021 |

**Table S2** Features of the tested primers for each PCR and nested-PCR

| **Primer sets** | | **Tm** | **Expected Size (bp)** | **Reference** | **Used by** | **Nested PCR: combination tested in this study** | **Gel profile** |
| --- | --- | --- | --- | --- | --- | --- | --- |
| PCR round 1 | F1:5’-CGACTTTTCGGAAGGGATGTATT-3’ | 59 | 550 | Mendonca & Arkush, 2004 | Andreou et al. 2012 Ercan et al 2015 Sana et al. 2017 | F2R2 F1R2 F2R1 | Multiband |
|  | R1: 5’-AGTCCCAAACTCGACGCACACT-3’ | 62 |  |  |  |  | Multiband |
| Nested | F2: 5’-CCCTCGGTTTCTTGGTGATTCATAATAACT-3’ | 64 | 434 |  |  |  | Multiband |
|  | R2: 5’-CTCGTCGGGGCAAACACCTC-3’ | 63 |  |  |  |  |  |
| PCR round 1 | F1: 5’-AATCGTATGACATTTTGTCGAC-3’ | 55 | 1115 | Gozlan et al. 2005 | Andreou et al. 2011 with modification Boitard et al. 2017 with modification | F2R2 F1R2 F2R1 | Multiband |
|  | R1: 5’-GAAGTCACAGGCGATTCGG-3’ | 59 |  |  |  |  | Multiband |
| Nested | F2: 5’-CAGGGCTTTTTAAGTCTT-3’ | 49 | 590 |  |  |  | Multiband |
|  | R2: 5’-TGATGGAGTCATAGAATTAACATCC-3’ | 58 |  |  |  |  |  |
| PCR round 1 | F1: 5’-ACAGGGCTTTTTAAGTCTTGT-3’ | 54 | 909 | Spikmans et al 2020* | - | F1R2 | Multiband |
|  | R1: 5’-GAAGTCACAGGCGATTCGG-3’ | 59 |  |  |  |  |  |
| Semi-nested | R2: 5’-ATGGAGTCATAGAATTAACATCC-3’ | 55 | 600 |  |  |  |  |
| PCR round 1 | F1: 5’-ACAGGGCTTTTTAAGTCTTGT-3’ | 54 | 600 | Spikmans et al 2020 | - |  |  |
|  | R2: 5’-ATGGAGTCATAGAATTAACATCC-3’ | 59 |  |  |  |  |  |
| Nested | F2: 5’-GCGGTAATTCCAGCTCCA-3’ | 57 | 154 | Current study: Cherif et al 2022 |  | ChF2 ChR2' | Monoband/Multiband |
|  | R2': 5’-CACTCAATTAAGCGCACACG-3’ | 58 |  |  |  |  |  |

*modification of the nested pcr of Gozlan et al. 2005


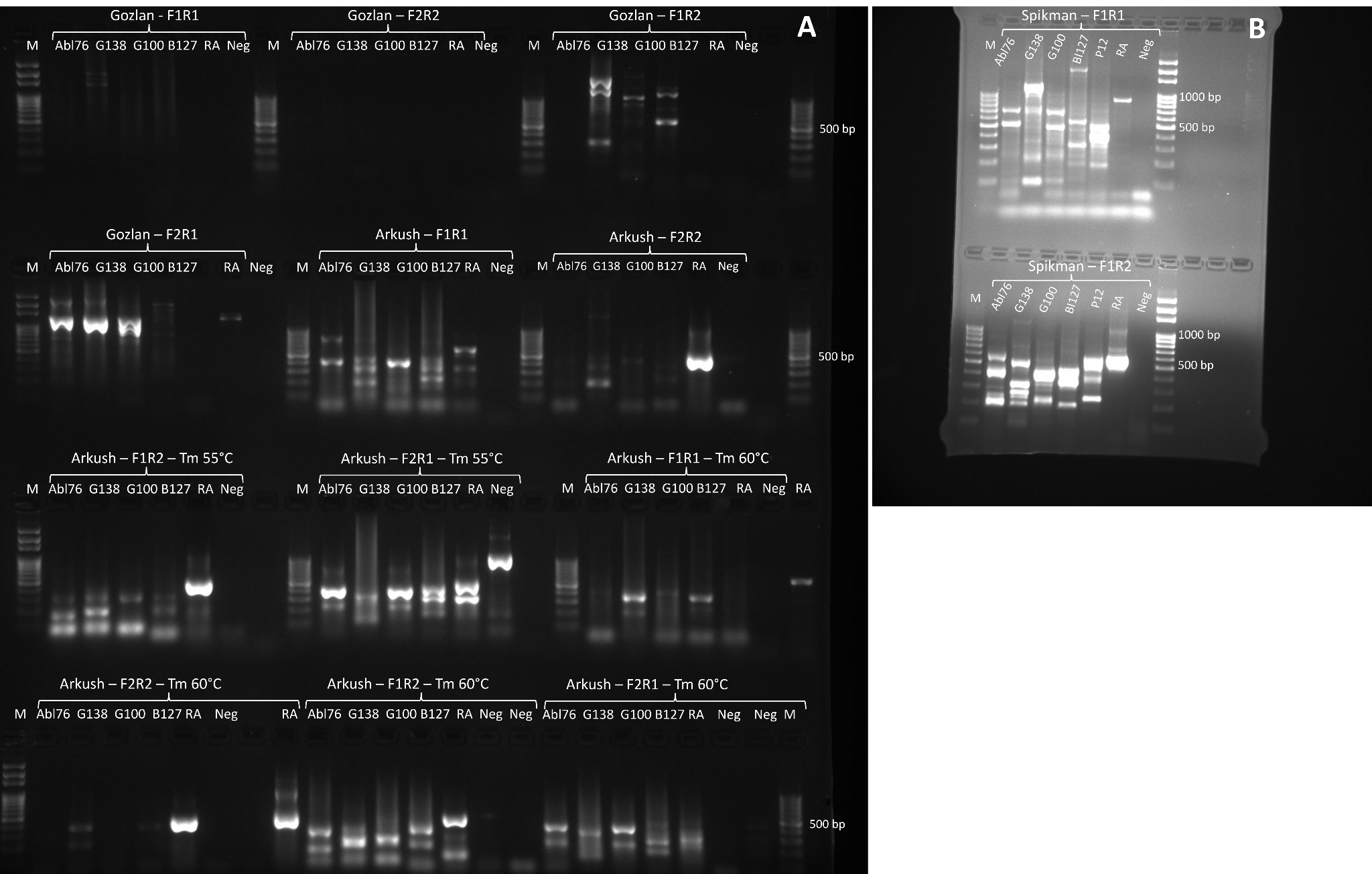


**Figure S1 Image of an agarose gel showing the published primers detection results of *S. destruens* extracted from five fish species using nested-PCR.** The origin of each PCR assay is indicated by the name of the author: **Gozlan =** primers from Gozlan et al. (2005); **Arkush =** primers from Mendonca & Arkush (2004); **Spikman =** primers from Spikmans et al. (2020). **P12**=*Pseudorasbora parva*, **B127**= *Rhodeus amarus*, **Abl76**= *Alburnus alburnus*, **G100**= *Rutilus rutilus*, G138= *Gobio gobio*, **RA**= positive control; Pure *S. destruens* DNA isolate RA-1. **Neg**= negative control. **M**= DNA Ladder. **F1R1** combination corresponds to the first PCR while the other combinations correspond to the nested PCR.


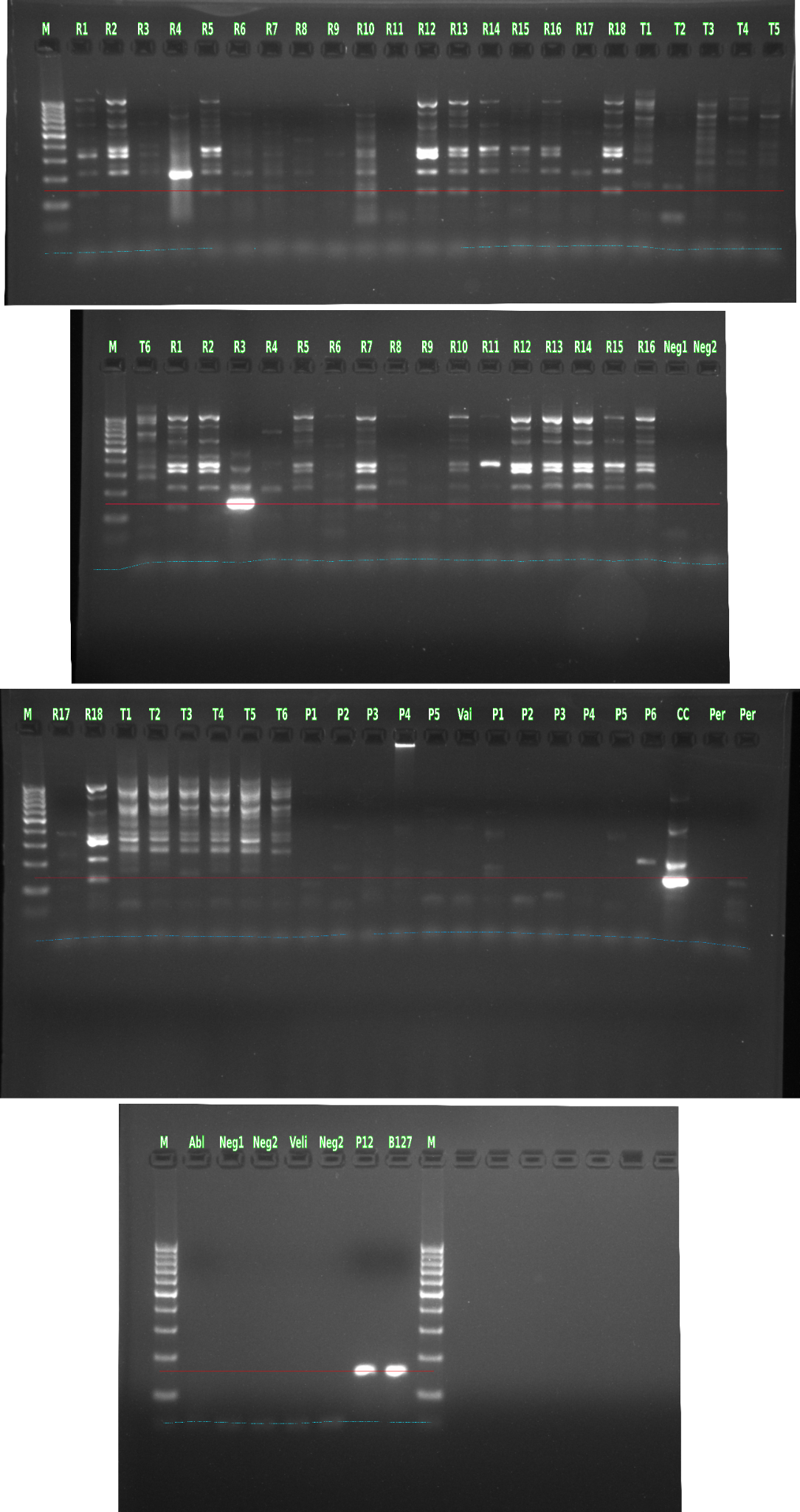


**Figure S2 Image of an agarose gel showing the detection results of *S. destruens* extracted from seven fish species using the newly designed nested-PCR. R1 to R16**= *Scardinius erythrophthalmus*, **T1 to T6**= *Salmo trutta*, **P1 to P6 and P12**=*Pseudorasbora parva*, **CC**= *Cyprinus carpio*, **Per**= *Perca fluviatilis*, **Vai**= *Phoxinus phoxinus*, **B127**= *Rhodeus amarus*, **Abl**= *Alburnus alburnus*, **Veli**= Cyprinus carpio brain cells Negative control, **Neg1 and 2**= no-template control. **M**= DNA Ladder. R1-R18 and T1-T6 are duplicated on the gel since fish organs were extracted using two different methods; the Macherey-Nagel Nucleospin Tissue DNA extraction kit and the Qiagen DNeasy PowerSoil Pro DNA extraction kit.
